# The Art of Healing
*Substance of a lecture by Professor Arthur Keith, M.D., at the Members' Conference of the Incorporated Society of Trained Masseuses, June 1920.


**Published:** 1920-07-17

**Authors:** 


### THE ART OF HEALING.*

Professor A. Keith in his opening words said he would little deserve the honour they had done him by asking him to come and speak to them unless he could say something which would serve to quicken their interests in their daily work. This Professor Keith must certainly have done after having delivered in a most lucid and interesting manner a lecture on the "Art of Healing," or, as he afterwards re-named it, "The Principles or Laws on which the Art of Healing is Based." The point which the lecturer brought home to his hearers was that the workers among the sick and injured did not by their skill actually heal the patients, but assisted Nature in doing so. Masseuses were in particular "healers"; but, whether they treated a case of sprained ankle or a broken wrist or a man suffering from sciatica or a woman suffering from rheumatic changes in the joints, their final success in dealing with them and the ultimate health of the patient would depend upon the knowledge of how far they (the masseuses) had grasped the principles which underlie the art of healing, and not upon their own particular art.

### A Property of the Flesh.

Professor Keith then pointed out that there were no parts of the body which could help us to understand the true nature of the art of healing better than the teeth. The teeth have no power of healing. Not even a dentist could assist in the healing of dental caries; all he could do would be to scrape out the caries and "stop" the tooth. If Nature had not endowed the other parts of our bodies with the power to heal neither the surgeon, physician, nor masseuse could have done anything for the cure of injury or disease; they could have done no more—perhaps not as much—as the dentist could for the teeth. The power of healing was a property in the flesh, and it was wrong to suppose that we did the healing; all we could do was to assist in the healing. The art which underlies the skill of the surgeon was the knowledge of how the tissues healed themselves.

Why was it that Nature did not supply the teeth with nerves? If the teeth had been supplied with nerves the patient would know at once if caries were present in the teeth. On the other hand, if nerves were present it would be far too painful to eat. The real reason why Nature had not endowed the teeth with the power of healing was that the substances had to be made so hard and resistant that it was impossible to introduce into the enamel or dentine the soft living units we speak of as cells, and it was in these cells that the power of healing lay. To understand the art of healing we must know something of the living units in which the power to heal resides. The cells furnished with the greatest degree of healing power were the white connective tissue cells. These cells, whenever a wound is made and the *débris* has been cleared away by other "workmen," immediately change their form, begin to divide and breed, and the gap which has been made is gradually filled up by these new connective tissue cells.

### Nature's Assistants.

It was John Hunter who observed that if a muscle or tendon was ruptured the patient ceased to have the power to call it into action. This loss of power was Nature's method of giving rest to the part, so that the cells could better do their work. Long after Hunter's time Pasteur and Lister discovered micro-organisms; this knowledge greatly improved the art of healing and taught the surgeons and others that cleanliness was essential in their art. The lecturer went on to say that the physician could by patient and combined observation strengthen the defenders (the white blood corpuscles) against these micro-organisms by the use of vaccines, antitoxins, and other methods of modern serumtherapy. In this case, again, the physician was not the healer: the power to heal was inherent in the patient's blood and body. The point brought home was that the power to heal, to regain health, lay not in the hands or heads of the surgeon, the physician, or masseuse, but in the living flesh and blood of the patient. Masseuses and other tenders of the sick were simply Nature's assistants, and to learn to wait on Nature judiciously was to produce the art of healing in its highest form.

### The Brain must Help.

Good results of treatment depend largely upon how far the brain is called in to help in healing. Quacks and Christian scientists realise this and make use of it. By bringing the patient's brain and central nervous system into play the reparative powers of the tissues are influenced for good or ill. If a masseuse knows the movements she applies to her patients, be they passive or active, friction or stroking, effleurage or petrisage, she assists the natural powers of the body to effect a cure, and you not only bring to bear on your patient the confidence of the faithhealer and the audacity of the quack, but the continued assurance which only rational knowledge can beget in a healer. A masseuse must have the knowledge of the power of reparation inherent in bodies of man and beast alike.

* Substance of a lecture by Professor Arthur Keith, M.D., at the Members' Conference of the Incorporated Society of Trained Masseuses, June 1920.

